# Nicotine‐induced neuroplasticity in striatum is subregion‐specific and reversed by motor training on the rotarod

**DOI:** 10.1111/adb.12757

**Published:** 2019-04-10

**Authors:** Valentina Licheri, Daniel Eckernäs, Filip Bergquist, Mia Ericson, Louise Adermark

**Affiliations:** ^1^ Addiction Biology Unit, Department of Psychiatry and Neurochemistry, Institute of Neuroscience and Physiology, The Sahlgrenska Academy University of Gothenburg Gothenburg Sweden; ^2^ Department of Pharmacology, Institute of Neuroscience and Physiology, The Sahlgrenska Academy University of Gothenburg Gothenburg Sweden

**Keywords:** amygdala, endocannabinoids, motor‐skill learning

## Abstract

Nicotine is recognized as one of the most addictive drugs, which in part could be attributed to progressive neuroadaptations and rewiring of dorsal striatal circuits. Since motor‐skill learning produces neuroplasticity in the same circuits, we postulate that rotarod training could be sufficient to block nicotine‐induced rewiring and thereby prevent long‐lasting impairments of neuronal functioning. To test this hypothesis, Wistar rats were subjected to 15 days of treatment with either nicotine (0.36 mg/kg) or vehicle. After treatment, a subset of animals was trained on the rotarod. Ex vivo electrophysiology was performed 1 week after the nicotine treatment period and after up to 3 months of withdrawal to define neurophysiological transformations in circuits of the striatum and amygdala. Our data demonstrate that nicotine alters striatal neurotransmission in a distinct temporal and spatial sequence, where acute transformations are initiated in dorsomedial striatum (DMS) and nucleus accumbens (nAc) core. Following 3 months of withdrawal, synaptic plasticity in the form of endocannabinoid‐mediated long‐term depression (eCB‐LTD) is impaired in the dorsolateral striatum (DLS), and neurotransmission is altered in DLS, nAc shell, and the central nucleus of the amygdala (CeA). Training on the rotarod, performed after nicotine treatment, blocks neurophysiological transformations in striatal subregions, and prevents nicotine‐induced impairment of eCB‐LTD. These datasets suggest that nicotine‐induced rewiring of striatal circuits can be extinguished by other behaviors that induce neuroplasticity. It remains to be determined if motor‐skill training could be used to prevent escalating patterns of drug use in experienced users or facilitate the recovery from addiction.

## Graphical abstract

This preclinical study shows that nicotine alters amygdalo‐striatal circuits in a distinct temporal and spatial sequence and impairs endocannabinoid signaling in a long‐lasting manner. However, intense motor‐skill training during the initial week of withdrawal completely prevents both acute and progressive neuroadaptations in the striatum. If a functional shift in amygdalo‐striatal circuits is a neurobiological underpinning of substance abuse liability, restoring neuronal function by motor‐skill training might be sufficient to suppress escalated patters of substance use.

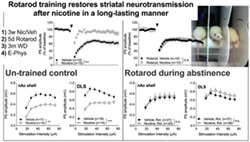

## 1 INTRODUCTION

Tobacco use is a leading preventable cause of death worldwide. Even though the use of conventional cigarettes has decreased during the last 20 years, smoking is still a major health problem. In addition, the introduction of electronic cigarettes (e‐cigarettes) has once again increased nicotine use globally, and there is an association between initial e‐cigarette use and cigarette smoking later in life.^1^ In order to reduce smoking prevalence, there is a need for more basic research that defines the effects by nicotine on neuronal circuits, and studies outlining how nicotine‐mediated maladaptation of neuronal function can be prevented.

Both clinical and preclinical studies have implicated the striatal nucleus in acute and long‐lasting effect by nicotine, and striatal circuit dysfunction has repeatedly been shown in dependent smokers.^2^, ^3^, ^4^, ^5^, ^6^, ^7^ The rodent striatum can be subdivided into nucleus accumbens (nAc) shell and core (linked to drug reinforcement), dorsomedial striatum (DMS; linked to reward‐guided behaviors), and dorsolateral striatum (DLS; linked to habitual responding).^8^, ^9^, ^10^, ^11^ Preclinical studies suggest that striatal circuits are progressively transformed during protracted drug taking, and that neurophysiological transformations are transferred and stored in the DLS, which may promote habitual/compulsive drug taking behavior (addiction).^2^, ^5^, ^12^, ^13^ Drug‐induced rewiring of dorsal striatum has furthermore been suggested to be driven by functional shifts in neuronal circuits of the amygdala.^14^ Even though a causal relationship between these neuronal transformations and the development of addiction has not been established, we speculate that interventions aimed at restoring neuronal function could be beneficial to improve smoking cessation.

Interestingly, during the acquisition and consolidation of a motor skill, striatal circuits are progressively rewired in a spatial and temporal manner that partially resembles the effects by nicotine. In fact, studies of motor‐skill consolidation have led up to the postulate that neuroadaptations initially occurring in the DMS are transferred to the DLS where they may become permanent.^9^, ^15^, ^16^ If nicotine‐induced striatal rewiring depends on antecedent activity in the DMS, motor‐skill learning on the rotarod might in this regard act to counterbalance nicotine‐induced neurophysiological transformations and/or prevent progressive neuroadaptations. Based on this line of reasoning, we hypothesize that a period of training on the rotarod would prevent the progressive rewiring of neuronal circuits and extinguish the cascade of neurophysiological adaptations elicited by nicotine.

## 2 MATERIALS AND METHODS

### 2.1 Experimental outline

In this study, we test the hypothesis that motor‐skill training on the rotarod is sufficient to block the temporal and spatial sequence of neuronal transformations that are elicited by nicotine. Group‐housed male rats (200‐220 g, n = 30 per round, with three rounds in total = 90 rats) were randomized to receive vehicle or nicotine injections over three consecutive weeks. Behavioral sensitization to the locomotor‐stimulatory properties of nicotine was assessed in an open‐field arena. Following behavioral sensitization, a subset of animals was trained for 5 days on the rotarod, resulting in a total of four groups of rats: vehicle control (n = 29), vehicle rotarod (n = 16), nicotine control (n = 29), and nicotine rotarod (n = 16). One set of animals was used to monitor locomotor behavior, and the rest were examined in electrophysiological recordings to explore progressive neuroadaptations in neuronal circuits of the striatum and amygdala.

### 2.2 Drugs and solutions

Nicotine tartrate was dissolved in 0.9% NaCl, adjusted to pH 7.2‐7.4 with NaHCO_3_ and administered at 1.0 mg/kg sc (0.36 mg/kg nicotine). Modified artificial cerebrospinal fluid (aCSF) contained (in mM): 220 sucrose, 2 KCl, 1.3 NaH_2_PO_4_, 6 MgCl_2_, 0.2 CaCl_2_, 26 NaHCO_3_, and 10 D‐glucose, while regular aCSF consisted of 124 NaCl, 4.5 KCl, 2 CaCl_2_, 1 MgCl_2_, 26 NaHCO_3_, 1.2 NaH_2_PO_4_, and 10 D‐glucose. The GABA_A_ receptor antagonist picrotoxin was dissolved in aCSF to 50 μM, while dopamine D2 receptor agonist quinpirole hydrochloride was dissolved in H_2_O to 80 mM and further diluted in aCSF to 5 μM. For whole‐cell recordings, the internal solution contained (in mM): 150 CsCl, 10 Hepes, 2 MgCl_2_, 0.3 Na_2_GTP, 3 MgATP, and 0.2 BAPTA, with pH adjusted to 7.2 with CsOH, and osmolarity set to 298 mOsm with sucrose. To isolate spontaneous inhibitory postsynaptic currents (sIPSCs), the NMDA receptor antagonist D‐(−)‐2‐Amino‐5‐phosphonopentanoic acid (APV) (50 μM) and the AMPA receptor antagonist CNQX (10 μM) were added to the aCSF. Drugs and solutions were purchased from Sigma‐Aldrich (Stockholm, Sweden) or Tocris (Abingdon, UK).

### 2.3 Animals

Male Wistar rats (Janvier, France) were group housed and kept on a 12/12 hours light/dark cycle at 20°C with 50% humidity with free access to food and water. All experiments were approved by the Gothenburg Animal Research Ethics Committee and conducted during the light time cycle.

### 2.4 Nicotine treatment and behavioral sensitization

The animals were randomly assigned to receive either nicotine or saline over a period of 3 weeks. Subcutaneous injections of nicotine were given five times a week (15 injections in total). To minimize the possible pain caused by acidic nicotine, pH was normalized using NaHCO_3_.^17^ Even though not self‐administered, this protocol has repeatedly been shown to induce robust behavioral sensitization to nicotine that is sustained for at least 7 months.^18^ Locomotor behavior was monitored after the first and last nicotine injection, and in a subset of animals 1 week after rotarod training. Vertical and horizontal movements were registered in an open‐field arena (40 × 40 cm, Med Assoc., Fairfax, VT, USA) placed in a sound attenuated, ventilated, and dim lit box. The open‐field arena was equipped with a two‐layer grid, consisting of rows of photocell beams, and consecutive beam breaks were tracked by a computer‐based system (Activity Monitor 7, Med Assoc., St. Albans, VT, USA). Rats were allowed to habituate to the box for 30 minutes and were then injected with nicotine or vehicle, after which locomotor behavior was registered for another 30 minutes.

### 2.5 Motor‐skill training on the rotarod

One week after nicotine/vehicle treatment, rats were trained to stay on an accelerating rotarod device (LE‐8500, Panlab S.L.U., Barcelona, Spain) placed in a ventilated and sound‐attenuated cupboard. The animals were trained to stay on the rotating rod as it accelerated from four to 40 rpm over 5 minutes. Each rat was trained for five consecutive days, with four trials per day, each day with an intersession interval of 10 minutes. The maximum time for each trial was set to 6 minutes. Following rotarod training, animals were either monitored in an open‐field arena (1 week after rotarod), or in ex vivo electrophysiology (3 to 7 days after rotarod, or 3 months after rotarod).

### 2.6 Brain slice preparation for ex vivo electrophysiology

For brain slice preparation, rats were anesthetized with isoflurane (Forene, Baxter, Sweden) before decapitation, and brains rapidly removed and submerged in continuously oxygenated (95% O_2_, 5% CO_2_) modified aCSF. Coronal brain slices (250 μm) were sectioned using a Leica VT 1200S Vibratome (Leica Microsystems AB, Bromma, Sweden) and were allowed to equilibrate at 34°C for 30 minutes in oxygenated normal aCSF before maintained in room temperature for the remainder of the day. The experimenters performing electrophysiological recordings were blind to drug treatment.

### 2.7 Electrophysiological field potential recordings

**Figure 1:**
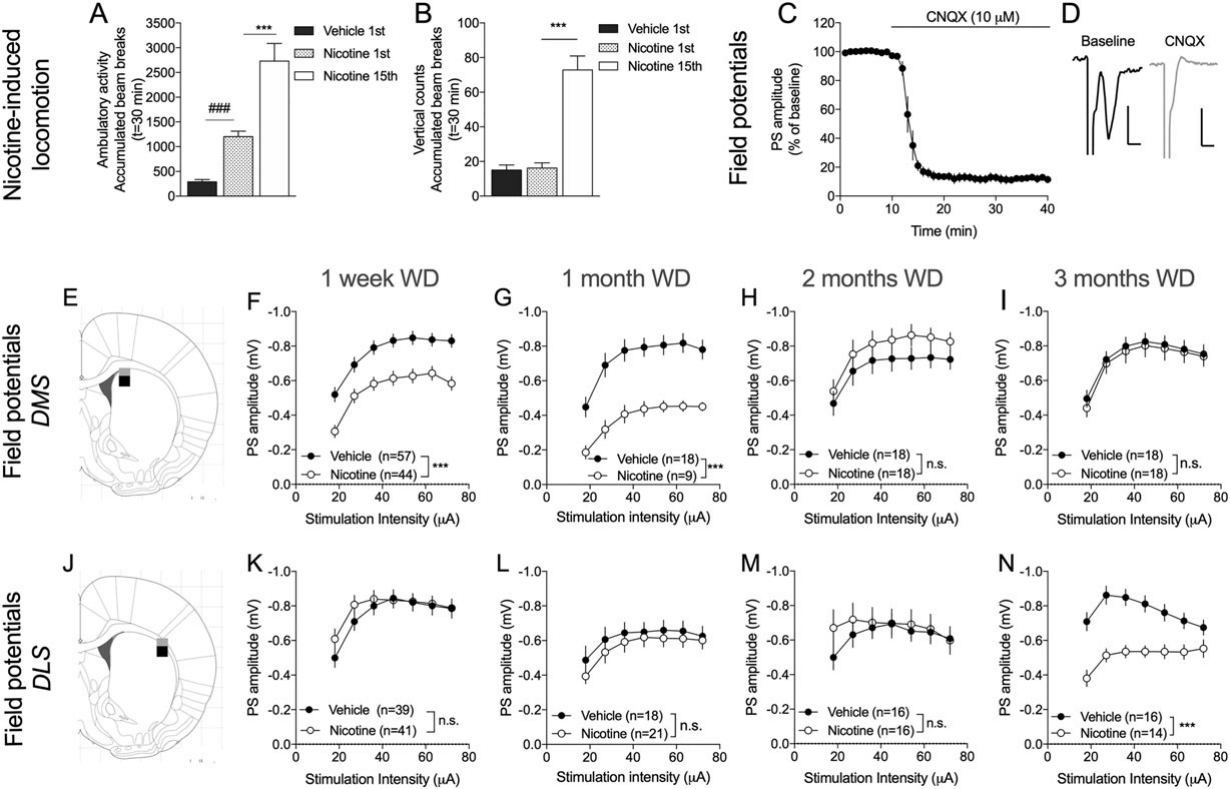
Nicotine produces behavioral sensitization and neurophysiological transformations. A, Nicotine significantly enhanced open‐field locomotion and repeated exposure produced behavioral sensitization. B, Rearing activity was not significantly enhanced by nicotine during the first exposure, but a significant increase in vertical movement was detected with repeated exposure. C, Electrophysiological field potential recordings demonstrated a robust depression elicited by the AMPA receptor antagonist CNQX on evoked field potentials. Calibration: 0.2 mV, 2 ms. D, Example traces show evoked PSs during baseline (black) and after CNQX perfusion (gray). Note that the prepulse remains while the PS disappears following CNQX perfusion. E, The black square represents the position of recording electrodes in the DMS, while the gray square shows the position of stimulation electrodes. F‐I, Field potential recordings revealed that brain slices from nicotine‐treated rats exhibited a depressed input/output function, which remained for 1 month after WD. J, The black square represents the position of recording electrodes in the DLS, while the gray square shows the position of stimulation electrodes. ‐N, Field potential recordings in the DLS showed no trend towards treatment effect with regards to input/output function during the first months of WD, but a significant depression arose with protracted abstinence. Data are mean values ± SEM. The number of animals is shown in A and B, while the numbers of recordings are displayed in F‐N. Individual datasets are based on at least five animals/treatment group. DLS, dorsolateral striatum; DMS, dorsomedial striatum; SEM, standard error of the mean; WD, withdrawal

Electrophysiological field potential recordings were performed as previously described.^19^ In brief, one hemisphere of the slice was constantly perfused with prewarmed aCSF (30°C, 2 mL/min), and field population spikes (PSs) were evoked with a stimulating electrode (monopolar tungsten electrode, World Precision Instruments, FL, USA, type TM33B, 20‐second interpulse interval) and registered with a recording electrode (outer diameter 1.5 μm, resistance ranging from 2.5 to 4.5 MΩ, World Precision Instruments, FL, USA) in subregions of the striatum and amygdala. Stimulating electrodes were placed close to the border of the DLS and the overlaying white matter while positioned locally in nAc, BLA, and CeA (Figures 1 and 3). Signals were amplified with a custom‐made amplifier, filtered at 3 kHz, and digitized at 8 kHz. PSs were evoked with increasing stimulation strength (18‐72 μA) to create an input/output function. When assessing the responsiveness to applied agonists/antagonists PS amplitude was set to half max response, and a stable baseline was recorded for 10 minutes before drugs were administered via bath perfusion. Endocannabinoid‐mediated long‐term depression (eCB‐LTD) was induced by four trains of 100 pulses delivered at 100 Hz with a 4‐second intertrain interval (HFS stimulation). This protocol has previously been shown to robustly induce eCB signaling.^20^

### 2.8 Whole‐cell recordings in voltage clamp mode

sIPSCs were recorded in striatal medium spiny neurons (MSNs) voltage clamped at −65 mV as previously described in detail.^21^ Slices were under constant flow of preheated aCSF (33‐34°C, 2 mL/min), and whole‐cell recordings were conducted with an Axopatch 700B amplifier (Axon Instruments, Foster City, CA, USA), filtered at 2 kHz, and digitized at 5 kHz. Only recordings with a stable series resistance that varied less than 20% and did not exceed 25 MΩ were included in the analysis. Data were acquired using Clampex 10.2 (Molecular devices, Axon CNS, CA, USA), and off‐line analysis performed manually using the parameters for sIPSC analysis in Minianalysis 6.0 (Synaptosoft, Decatur, GA, USA). Amplitude, frequency, time to rise, and time to decay were calculated as an average of the events observed within the 3 minutes of recording.

### 2.9 Statistics

All data were analyzed using Clampfit 10.2, Microsoft Excel, and GraphPad Prism 7 (GraphPad Software, San Diego, CA, USA). Gaussian distribution was tested with D'Agostino‐Pearson omnibus normality test. For behavioral and electrophysiological data repeated measure, two‐way analysis of variance (ANOVA) was used for comparisons over time, and input/output function, while paired or unpaired *t* tests and Kolmogorov‐Smirnov test were used when applicable. All parameters are given as mean ± standard error of the mean (SEM), and the level of significance was set to *P* < .05.

## 3 RESULTS

### 3.1 Repeated nicotine administration produces behavioral sensitization and neurophysiological transformations in dorsal striatal subregions

Rats were challenged with vehicle or nicotine for 15 days and sensitization towards the locomotor‐stimulatory properties of nicotine was seen consistently in all nicotine‐treated animals (vehicle 1st vs nicotine 1st: *t*_52_ = 7.64, *P* < .001; nicotine 1st vs nicotine 15th: *t*_26_ = 4.13, *P* < .001) (Figure 1A). Rearing activity was not modulated by acute nicotine administration (*t*_52_ = 0.28, *P* = .79) but significantly enhanced following additional treatment (nicotine 1st vs nicotine 15th: *t*_26_ = 7.49, *P* < .001) (Figure 1B).

Electrophysiological field potentials recordings were performed in dorsal striatal subregions after 1 week and 1 to 3 months of nicotine withdrawal. Field potentials were instantly suppressed by CNQX (10 μM) (Figure 1C‐D), indicating that these recordings primarily reflect activation of AMPA receptors (*t*_6_ = 28, *P* < .001). In the DMS, nicotine‐treated rats exhibited a decline in input/output function, which remained for up to 1 month of withdrawal (1 week: *F*_1,99_ = 18.6, *P* < .001; 1 month: *F*_1,26_ = 19.3, *P* = .002; 2 months: *F*_1,33_ = 1.46, *P* = .24; 3 months: *F*_1,34_ = 0.14, *P* = .71) (Figure 1F‐I). In the DLS, on the other hand, a change in input/output function was apparent first after 3 months withdrawal (1 week: *F*_1,78_ = 0.33, *P* = .57; 1 month: *F*_1,36_ = 0.51, *P* = .48; 2 months: *F*_1,30_ = 0.23, *P* = .64; 3 months: *F*_1,28_ = 22.3, *P* < .001) (Figure 1K‐N). There was no significant effect by nicotine treatment on synaptic depression induced by the dopamine D2 receptor agonist quinpirole, neither after 1 week withdrawal (DMS: *F*_1,19_ = 1.84, *P* = .19; DLS: *F*_1,22_ = 0.53, *P* = .49) (Figure S1) nor after 3 months withdrawal (DMS: *F*_1,35_ = 2.28, *P* = .14; DLS: *F*_1,26_ = 2.26, *P* = .15).

**Figure 2:**
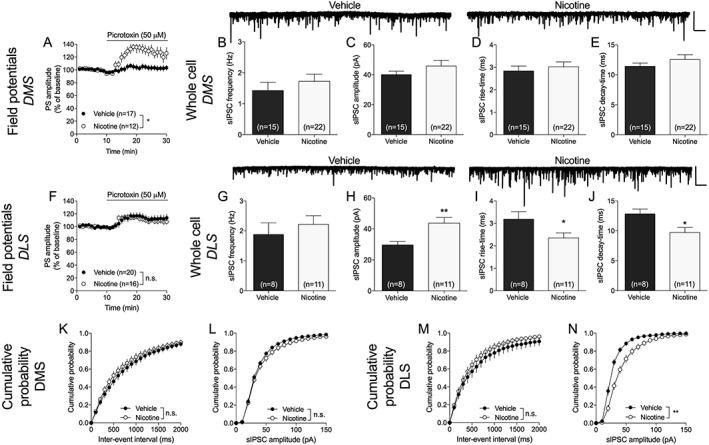
Nicotine elicits a region‐specific modulation of GABAergic neurotransmission. A, Disinhibition induced by the GABA_A_ receptor antagonist picrotoxin was significantly enhanced in the DMS of nicotine‐exposed animals. B‐E, Whole‐cell recordings revealed no treatment effects on recorded sIPSCs in the DMS. Example traces show recorded sIPSCs in vehicle and nicotine‐treated rats. Scale bar is 200 pA and 10 s. F, There was no effect by nicotine treatment on picrotoxin‐induced disinhibition in the DLS. G‐J, In the DLS, recordings performed in brain slices from nicotine‐exposed animals showed an increase in sIPSC amplitude, while rise and decay time was depressed. K‐N, Nicotine had no effect on the cumulative distributions of either event intervals or amplitudes in the DMS, while a significant effect on amplitude was present in the DLS. Example traces show recorded sIPSCs in vehicle‐ and nicotine‐treated rats. Scale bar is 200 pA and 10 s. Data are mean values ± SEM; n = the number of recordings. Individual datasets are based on at least five animals/treatment group. DLS, dorsolateral striatum; DMS, dorsomedial striatum; SEM, standard error of the mean; sIPSC, spontaneous inhibitory postsynaptic current

Field potentials primarily reflect excitatory neurotransmission, and to assess changes in GABAergic neurotransmission, disinhibition induced by the GABA_A_ receptor antagonist picrotoxin (50 μM), and sIPSCs were studied. One week after the last drug exposure recordings performed on nicotine‐exposed animals revealed a significant increase in picrotoxin‐induced disinhibition in the DMS (*F*_1,24_ = 10.1, *P* = .004) (Figure 2A), but not in the DLS (*F*_1,34_ = 0.13, *P* = .72) (Figure 2F). Whole‐cell recording performed in voltage clamp mode showed no significant effects by nicotine treatment on recorded sIPSCs in the DMS (frequency: *t*_35_ = 0.92, *P* = .36; amplitude: *t*_35_ = 1.13, *P* = .27; rise: *t*_35_ = 0.62, *P* = .53; decay: *t*_35_ = 1.04, *P* = .30) (Figure 2B‐E), while sIPSC amplitude was significantly modulated in the DLS (frequency: *t*_17_ = 0.71, *P* = .48; amplitude: *t*_17_ = 3.06, *P* = .007; rise: *t*_17_ = 2.13, *P* = .048; decay: *t*_17_ = 2.54, *P* = .02) (Figure 2G‐J). Analyzing cumulative probability of recorded events supported a selective effect by nicotine on sIPSC amplitude in the DLS (Kolmogorov‐Smirnov test: DMS: frequency: D = 0.06, *P* = 1.0; amplitude: D = 0.26, *P* = .09. DLS: frequency: D = 0.29, *P* = .15; amplitude: D = 0.39, *P* = .002) (Figure 2K‐N). One month after nicotine exposure, there were no effects by treatment on recorded sIPSCs in neither DMS (frequency: *t*_15_ = 1.27, *P* = .23; amplitude: *t*_15_ = 1.16, *P* = .26; rise time: *t*_15_ = 0.36, *P* = .72; decay time: *t*_15_ = 0.88, *P* = .39) nor DLS (frequency: *t*_17_ = 0.25, *P* = .80; amplitude: *t*_17_ = 0.06, *P* = .95; rise time: *t*_17_ = 0.33, *P* = .74; decay time: *t*_17_ = 0.73, *P* = .48) (Figure S2).

### 3.2 Temporal rewiring also involves other circuits of the striatal and amygdala nucleus

**Figure 3:**
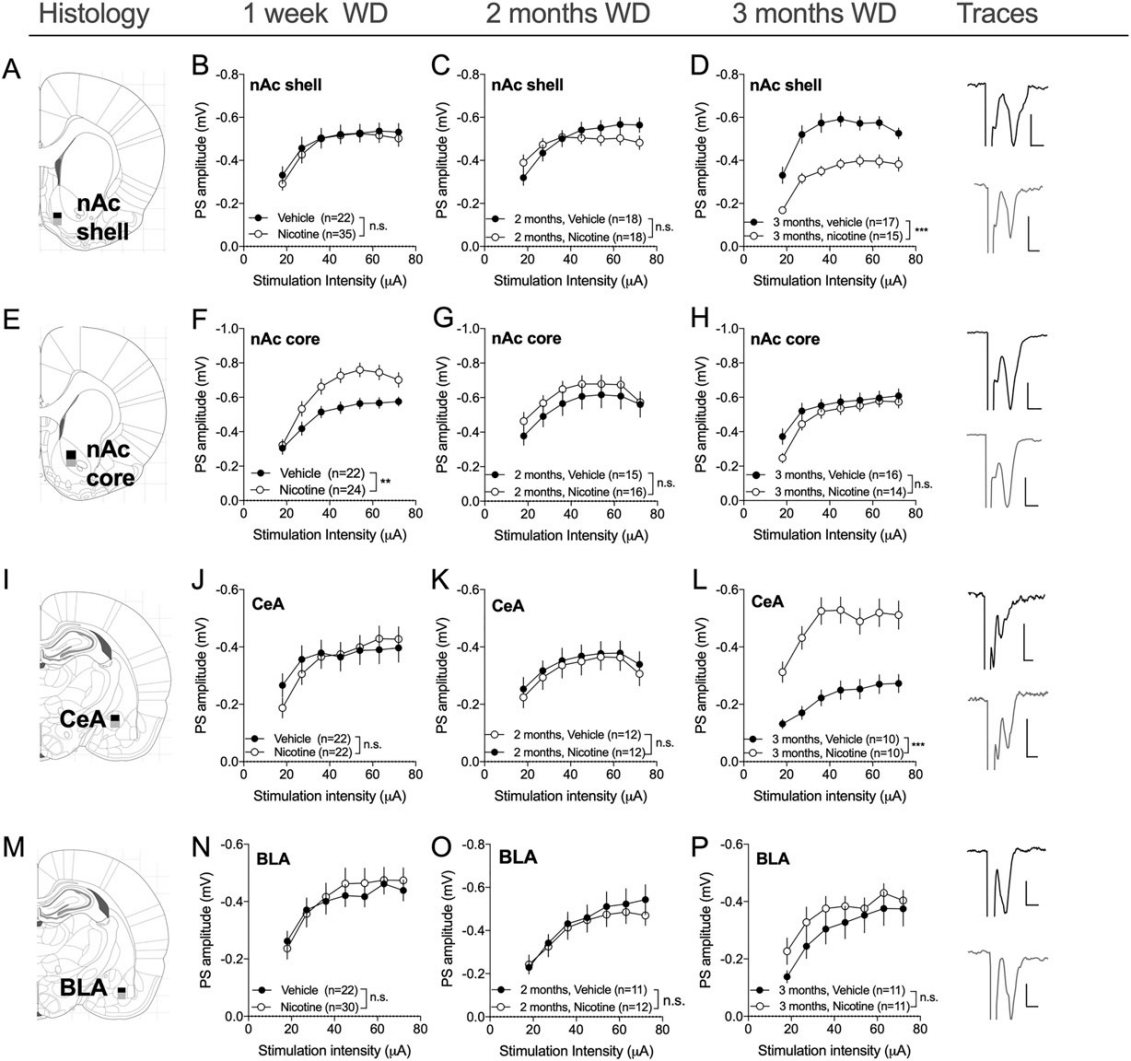
Nicotine produces neuroadaptations in a spatial and temporal manner. A, Schematic drawing showing the region for nAc shell recordings. B‐D, Recordings performed in the nAc shell demonstrated a decline in input/ouput function in brain slices from nicotine‐exposed animals that was established after 3 months WD. E, Schematic drawing showing the area for nAc core recordings. F‐H, In nAc core, input/ouput was initially increased but returned to baseline during protracted WD. I, Schematic drawing showing the region for CeA recordings. J‐L, Neuroadaptations in the CeA exhibited a similar time course as transformations recorded in nAc shell and DLS, and an increased input/output function was detected after 3 months WD. M, Schematic drawing showing the region for BLA recordings. N‐P, Nicotine did not modulate input/output function in the BLA at any time point analyzed. Example traces displayed to the right correspond to evoked PSs 3 months after exposure to vehicle (black) or nicotine (gray). Scale bar: 0.2 pA, 2 ms. For histological presentations, black squares represent the position of recording electrodes, while gray squares correspond to the placement of stimulation electrodes. Data are mean values ± SEM; n = the number of recordings. Individual datasets are based on at least five animals/treatment group. BLA, basolateral amygdala; CeA, central nucleus amygdala; nAc, nucleus accumbens; SEM, standard error of the mean; WD, withdrawal

Striatal neuroplasticity has been reported following exposure also to other drugs of abuse, and it is possible that rewiring of striatal circuits could be a common denominator during the establishment of addiction. Importantly, studies evaluating neuroadaptations elicited by cocaine suggest that striatal rewiring is linked to parallel transformations in ventral striatum and neuronal circuits of the amygdala.^14^ To assess changes in other brain subregions that could be involved in establishing nicotine‐induced rewiring of dorsal striatum, neurotransmission was recorded in ventral striatum (nAc shell and nAc core) and amygdala (basolateral amygdala [BLA] and central nucleus amygdala [CeA]). During the acute phase of withdrawal, input/output function was significantly increased in the core region of the nAc (*F*_1,45_ = 9.49, *P* = .004), but not in nAc shell (*F*_1,55_ = 0.01, *P* = .92), or CeA (*F*_1,42_ = 0.02, *P* = .89). After 2 months abstinence, there were no significant effects on input/output function in any brain subregion tested (shell: *F*_1,34_ = 0.18, *P* = .67; core: *F*_1,29_ = 0.76, *P* = .39; CeA: *F*_1,22_ = 0.15, *P* = .71), but robust changes were established in nAc shell and CeA at 3 months withdrawal (shell: *F*_1,30_ = 27, *P* < .001; CeA: *F*_1,18_ = 22, *P* < .001) (Figure 3). Nicotine did not affect input/output function in the BLA at any time point measured (1 week: *F*_1,49_ = 0.07, *P* = .79; 2 months: *F*_1,21_ = 0.14, *P* = .72; 3 months: *F*_1,20_ = 0.94, *P* = .34) (Figure 3).

### 3.3 Motor‐skill training on the rotarod elicits neurophysiological transformations

**Figure 4:**
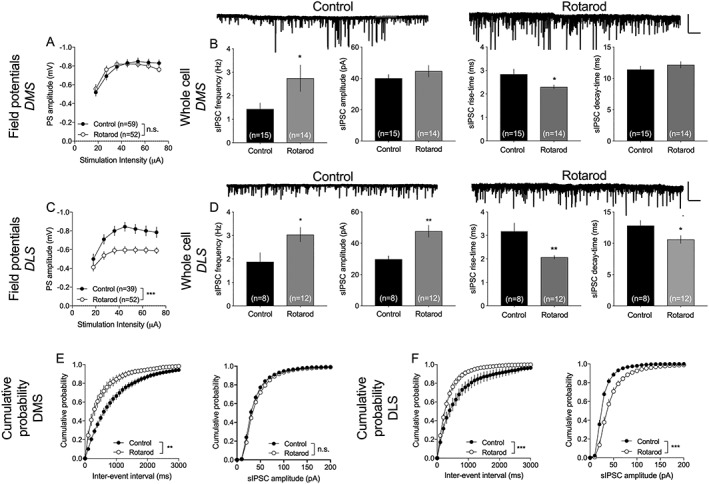
Training on the rotarod produces neurophysiological transformations. A, Field potential recordings performed in the DMS showed no effect of rotarod training on input/output function. B, Five days of motor‐skill training on the rotarod resulted in increased frequency and reduced rise time of recorded sIPSCs in the DMS. Example traces show recorded sIPSCs in control and rotarod‐trained rats. Scale bar is 200 pA and 10 s. C, In the DLS, input/output function was significantly depressed in brain slices from rats trained on the rotarod. D, Whole‐cell recordings revealed a significant effect by training on sIPSC amplitude, rise time, and decay time, while sIPSC frequency remained unaffected. E, Rotarod training significantly modulated the cumulative distribution of interevent intervals but had no effect on recorded amplitudes in the DMS. F, In DLS, rotarod affected the cumulative distributions of both event intervals and amplitudes. Example traces show recorded sIPSCs in control and rotarod‐trained rats. Scale bar is 200 pA and 10 s. Data are mean values ± SEM; n = the number of recordings, based on at least four animals/treatment group. DLS, dorsolateral striatum; DMS, dorsomedial striatum; sIPSC, spontaneous inhibitory postsynaptic current; SEM, standard error of the mean

Input/output function was not significantly altered in the DMS after rotarod training (*F*_1,109_ = 2.33, *P* = .13), but depressed in the DLS (*F*_1,89_ = 11.8, *P* < .001) (Figure 4A,C). At the same time, whole‐cell recordings showed an enhanced frequency of inhibitory inputs to MSNs in both subregions (frequency: DMS: *t*_27_ = 2.19, *P* = .03; DLS: *t*_18_ = 2.35, *P* = .03), while amplitude was selectively increased in DLS (amplitude: DMS: *t*_27_ = 1.05, *P* = .30; DLS: *t*_18_ = 3.58, *P* = .002) (Figure 4). sIPSC rise time was significantly reduced in both subregions (DMS: *t*_27_ = 2.22, *P* = .03; DLS: *t*_18_ = 3.86, *P* = .001), with decay time selectively altered in the DLS (DMS: *t*_27_ = 0.96, *P* = .34; DLS: *t*_18_ = 2.16, *P* = .04) (Figure 4). Analyzing cumulative probability of recorded events indicated an even more pronounced effect by rotarod training on sIPSC parameters, and a significant effect on interevent interval also in the DLS (Kolmogorov‐Smirnov test: DMS: frequency: D = 0.37, *P* = .002; amplitude: D = 0.27, *P* = .10. DLS: frequency: D = 0.55, *P* < .001; amplitude: D = 0.63, *P* < .001) (Figure 4E,F). Rotarod training also suppressed input/output in nAc shell (*F*_1,42_ = 7.75, *P* = .008), while nAc core (*F*_1,40_ = 0.05, *P* = .82), BLA (*F*_1,51_ = 0.42, *P* = .52), and CeA (*F*_1,44_ = 0.87, *P* = .36) remained unaffected (Figure S3). Synaptic depression induced by the dopamine D2 receptor agonist quinpirole (5 μM) was not modulated (DMS: *F*_1,22_ = 2.67, *P* = .17; DLS: *F*_1,22_ = 1.43, *P* = .24) (Figure S1).

### 3.4 Rotarod training abolishes nicotine‐induced rewiring of striatal circuits

**Figure 5:**
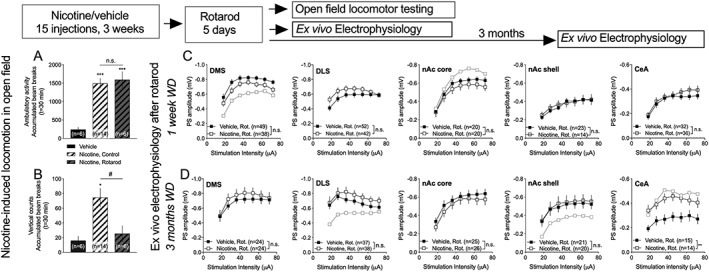
Rotarod training prevents neurodaptations in the striatum. A,B, Training on the rotarod did not affect the sensitized response to the locomotor‐stimulatory properties of nicotine but significantly reduced nicotine‐induced rearing. C, Ex vivo electrophysiological recordings performed on rotarod‐trained rats demonstrated no significant effects by nicotine treatment on input/output function in any brain region studied. For comparison, in brain regions where nicotine significantly modulated input/output function as compared with vehicle‐exposed control, the input/output function in untrained rats exposed to nicotine is indicated by an additional curve in gray. D, Following 3 months withdrawal, input/output function remained unaltered in striatal subregions, but an increase in evoked PSs was still present in CeA. Curves in gray represent input/output function in untrained rats exposed to nicotine 3 months earlier. Data are mean values ± SEM; n = the number of recordings, based on at least five animals/treatment group. CeA, central nucleus amygdala; DLS, dorsolateral striatum; DMS, dorsomedial striatum; nAc, nucleus accumbens; PS, population spike; SEM, standard error of the mean; WD, withdrawal

Vehicle and nicotine‐treated rats were trained for 5 days on the rotarod. In one set of rats, the ability for rotarod training to reverse behavioral sensitization was assessed. Rotarod training did not affect behavioral sensitization towards the locomotor‐stimulatory properties of nicotine as monitored in an open‐field arena (horizontal activity: Tukey's multiple comparisons test: vehicle vs nicotine; q = 8.23, *P* < .001; vehicle vs nicotine 1 month; q = 7.70, *P* < .001; nicotine vs nicotine 1 month: q = 0.66, *P* > .05) (Figure 5A). However, a sensitized response to the rearing‐stimulatory response was no longer present when comparing nicotine‐treated control rats with nicotine‐treated rats trained on the rotarod (Tukey's multiple comparisons test: vehicle vs nicotine; q = 4.45, *P* = .012; vehicle vs nicotine + rotarod; q = 0.61, *P* = .90; nicotine vs nicotine + rotarod: q = 3.73, *P* = .038) (Figure 5B).

Training on the rotarod furthermore prevented the nicotine‐induced increase in picrotoxin‐mediated disinhibition (DMS: *F*_1,20_ = 1.59, *P* = .22; DLS: *F*_1,22_ = 1.89, *P* = .18) and changes in sIPSCs caused by nicotine in the DLS (DMS: frequency: *t*_27_ = 2.28, *P* = .03; amplitude: *t*_27_ = 0.92, *P* = .36; rise time: *t*_27_ = 0.52, *P* = .60; decay time: *t*_27_ = 0.14, *P* = .88: DLS: frequency: *t*_17_ = 1.00, *P* = .33; amplitude: *t*_17_ = 0.52, *P* = .61; rise time: *t*_17_ = 0.35, *P* = .73; decay time: *t*_17_ = 0.48, *P* = .64) (Figure S4). In addition, nicotine‐induced suppression of input/output function in DMS (*F*_1,86_ = 3.60, *P* = .061), as well as nicotine‐induced transformations in nAc core were blocked during the acute phase of withdrawal (*F*_1,38_ = 1.95, *P* = .17) (Figure 5). In line with recordings from untrained animals, input/output function in DLS (*F*_1,92_ = 2.07, *P* = .15), nAc shell (*F*_1,35_ = 0.05, *P* = .83), and CeA (*F*_1,46_ = 0.47, *P* = .50) was not modulated during the acute phase of withdrawal (Figure 5). Following extended withdrawal, training on the rotarod 3 months earlier completely blocked nicotine‐induced transformations in DLS (*F*_1,73_ = 1.18, *P* = .28) and nAc shell (*F*_1,39_ = 0.08, *P* = .78). The increase in input/output function in the CeA, however, sustained (*F*_1,27_ = 8.74, *P* = .006) (Figure 5).

### 3.5 Training on the rotarod rescues synaptic plasticity in the form of eCB‐LTD

**Figure 6:**
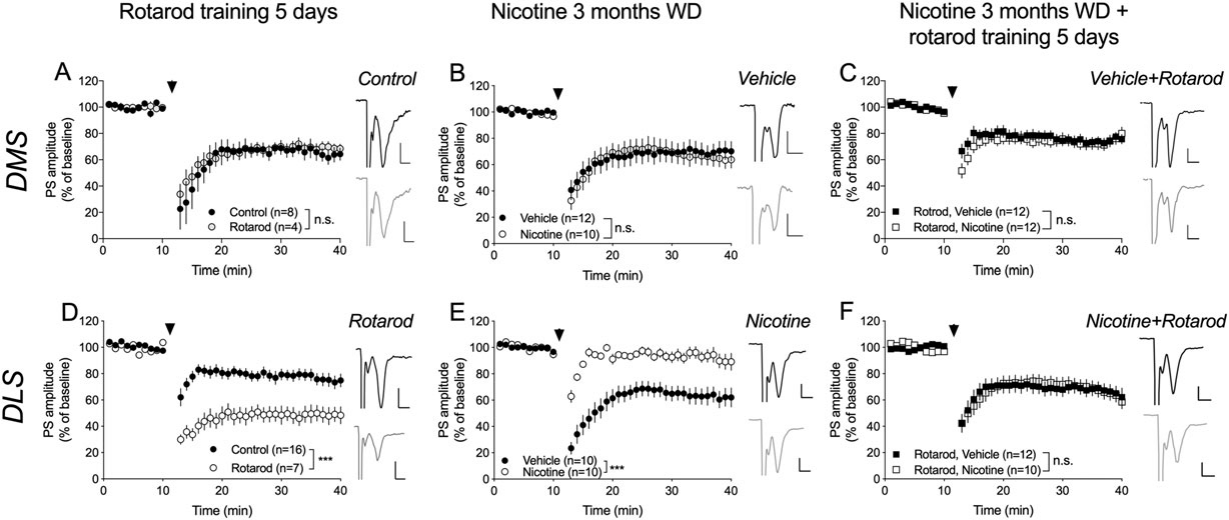
Rotarod training restores synaptic plasticity in the form of eCB‐LTD. A, Rotarod training did not affect eCB‐LTD induced by HFS in the DMS. B,C, Nicotine treatment did not modulate eCB‐LTD. D, Rotarod training facilitated eCB‐LTD in the DLS. E, Extended withdrawal inhibited synaptic depression in the DLS. F, Five days of rotarod training during withdrawal restored HFS‐induced LTD in DLS. Example traces shows evoked PS amplitudes at baseline (black) and after HFS (gray) for different treatment groups. Calibration: 0.2 mV, 2 ms. Data are mean values ± SEM; n = the number of recordings, based on at least five animals/treatment group. DLS, dorsolateral striatum; DMS, dorsomedial striatum; eCB‐LTD, endocannabinoid‐mediated long‐term depression; PS, population spike; SEM, standard error of the mean; WD, withdrawal

The eCB system plays a key role in mediating nicotine‐induced neuroplasticity, and nicotine has previously been shown to produce maladaptive eCB signaling that may have relevance for maintenance of addiction‐related behavior.^22^ In the last set of experiments, acute and long‐lasting effects by nicotine and rotarod training on eCB‐LTD were assessed in the DMS and DLS. During acute withdrawal, there was no effect by nicotine on eCB‐LTD (DMS: *F*_1,18_ = 0.01, *P* = .93; DLS: *F*_1,25_ = 0.39, *P* = .54), but a selective impairment was found in the DLS following protracted withdrawal (3 months withdrawal, vehicle vs nicotine‐treated: DMS: *F*_1,20_ = 0.00, *P* = .95; DLS: *F*_1,18_ = 15.8, *P* < .001) (Figure 6B,E). Recordings performed on brain slices from rats trained on the rotarod showed a significant facilitation of HFS‐induced eCB‐LTD in the DLS, but not in the DMS (DMS: *F*_1,10_ = 0.10, *P* = .76; DLS: *F*_1,21_ = 37, *P* < .001) (Figure 6A,D). Furthermore, training on the rotarod during the acute phase of withdrawal restored eCB‐LTD in nicotine‐exposed rats when assessed after 3 months later (vehicle vs nicotine: DMS: *F*_1,34_ = 0.27, *P* = .60; DLS: *F*_1,40_ = 0.27, *P* = .61) (Figure 6C,F).

## 4 DISCUSSION

The prevalence of e‐cigarette use has risen rapidly since the introduction 2007, especially among youths. There is a belief that the harmful component from smoking is the combustion of tobacco, and that tobacco free nicotine, which is used in e‐cigarettes, is safe. In the study presented here, the pure action of nicotine on neuronal circuits was assessed. It is important to note that nicotine was neither self‐administered nor smoked, and this experimental setup does thereby not resemble normal human smoking. At the same time, it is noteworthy that in despite of being unvoilational, pure nicotine exposure is sufficient to produce long‐lasting behavioral transformations that are accompanied by brain region‐specific transformations. These findings thereby support a potent effect by nicotine on biological systems and suggest that nicotine may produce prolonged reorganizations of neuronal circuits independent of route of administration or motivational aspects of drug taking.

Even though nicotinic acetylcholine receptors (nAChRs) are not expressed on striatal medium spiny projection neurons (MSNs), nicotine exposure exerts a complex modulatory effect on striatal neurotransmission.^21^ Furthermore, the data presented here show that repeated exposure to nicotine produce neuronal transformations in a distinct temporal and spatial sequence. We also show that neuroadaptations in DLS, nAc shell, and CeA occur at the same time point, which might be fundamental for establishing maladaptive incentive habits.^8^ If rewiring of amygdalo‐striatal circuits is a neurobiological underpinning of substance abuse liability, restoring neuronal function in these regions might be sufficient to suppress escalated patterns of substance use and to reinstate the control over behavior. Encouragingly, we found that a short period of training on the rotarod is sufficient to extinguish striatal neuroadaptations, both acutely and in a long‐lasting manner. We furthermore show that rotarod training restores synaptic plasticity in the form of eCB‐LTD. The eCB system has been reported to be impaired not only after nicotine exposure but also following exposure to other drugs of abuse,^22^, ^23^, ^24^, ^25^ and a maladaptive eCB system has been suggested to contribute to the development and maintenance of addiction‐related behavior.^26^ The finding that rotarod training is sufficient to restore eCB‐mediated plasticity thus further implicates a potential impact by behaviors that induce neuroplasticity on the treatment of addiction. If these theories hold true, motor‐skill training combined with exercise could be a valuable addition to pharmacological and psychosocial interventions when reducing not only smoking but also substance use in experienced drug users.

The datasets presented here demonstrates a spatially and temporally specific suppression of evoked field potentials in animals treated with nicotine, where the DMS (reward driven behaviors) is initially modulated while DLS (habitual responding) is recruited at a later stage. Even though inhibitory synaptic activity was not significantly modulated in the DMS of nicotine‐treated rats, picrotoxin produced a greater disinhibition, indicating that tonic inhibition might be enhanced following nicotine administration, thereby putatively suppressing evoked field potentials. Changes in input/output function lasted for 1 month in DMS and nAc core, and after 2 months, there were no detectable effect on neurotransmission in any brain region analyzed. At this time point, it is possible that other structures, such as cortical or dopaminergic areas in the midbrain, are recruited as an intermediate state before neurophysiological alterations arise in the striatum and amygdala.^11^ Following 3 months of abstinence from nicotine, robust transformations were established in the DLS, nAc shell, and CeA. These findings are especially interesting considering that previous studies, evaluating incubation of cocaine craving, reported a progressive increase in BDNF levels that peak in nAc and amygdala after 3 months of withdrawal.^27^ It is thus possible that progressive shifts in amygdalo‐striatal circuits not only contributes to the formation of incentive habits but also are linked to cue‐induced craving and relapse after prolonged abstinence.^8^, ^28^ Furthermore, at this time point, there is an increase in exploratory/anxiolytic behavior, which also appears to be directly linked to neurophysiological transformations in nAc shell.^5^

Previous studies have shown that dorsal striatum is recruited during consolidation of a motor skill, and that region‐ and pathway‐specific plasticity sculpts striatal circuits as the motor skill become automatized.^15^, ^16^ Electrophysiological recordings, performed in vivo and ex vivo, show an increase in MSN activity in the DMS during the initial training period, with the DLS being affected first when the skill is fully consolidated.^16^ In our hands, 5 days of rotarod training significantly increased sIPSC frequency in both the DMS and DLS and increased sIPSC amplitude in the DLS, indicating that presynaptic and postsynaptic transformations occur in a brain subregion‐specific manner. In addition, input/output function was significantly depressed in the DLS and nAc shell, suggesting that other brain regions may be recruited during this process. It should be noted that these recordings were performed 3 to 7 days after the last training session, and we do not know if these transformations are transient or long lasting. Furthermore, these studies do not separate between MSNs expressing dopamine D1 and D2 receptors, which also might affect the experimental outcome.^29^ Nevertheless, the data presented here implicate a selectivity with regard to rotarod‐elicited neurophysiological transformations, which could be important for understanding how the formation of motor‐related memories initially involves recruitment the DMS and how it is then transferred and stored in the DLS.

Progressive rewiring of dorsal striatum has been reported following exposure to several types of drugs of abuse and might be driven by neuroplasticity in other circuits of the striatal and amygdala nucleus.^14^ In agreement with these theories, we found that nicotine acutely modulates neurotransmission in nAc core, with protracted withdrawal resulting in altered input/output function in nAc shell and CeA. However, in contrast to what has been reported after cocaine self‐administration, nicotine did not produce a significant effect on neurotransmission in BLA.^14^ It is possible that parallel neuroplasticity in the nAc shell and DLS underlies the reduced control over incentive habits and might in this regard play a role for the transition from recreational drug use towards addiction. Importantly, 5 days of rotarod training during the initial week of withdrawal completely prevented not only the acute transformations in DMS and nAc core but also progressive neuroadaptations in DLS and nAc shell. Furthermore, we found that the sensitized response to nicotine‐induced rearing, which previously has been linked to DMS, was blocked in animals trained on the rotarod.^2^ These findings collectively suggest that motor‐skill learning not only reverses nicotine‐induced neuroadaptations in the acute phase but also prevents the progressive rewiring of striatal circuits that normally develop during withdrawal after nicotine use.

## CONFLICT OF INTEREST

The authors report no financial disclosure or potential conflicts of interest.

## AUTHOR CONTRIBUTIONS

LA designed the study, performed field potential recordings, data analysis, and wrote the manuscript. VL performed whole‐cell recordings, data analysis, and edited the manuscript. DE performed rotarod training, data analysis, and edited the manuscript. FB contributed during planning of the experiments, interpretation of the data, and preparation and writing of the manuscript. ME assisted during planning and execution of behavioral test and edited the manuscript.

## Supporting information


**Figure S1.** **Synaptic depression induced by dopamine D2 receptor activation is not significantly modulated by nicotine or rotarod‐training.** A, C) There was no effect by previous nicotine‐treatment on the responsiveness to the dopamine D2 receptor agonist quinpirole. B, D) Five days of rotarod‐training was not sufficient to modulate quinpirole‐induced synaptic depression in dorsal striatum. Data are mean values ± SEM. n = the number of recordings. Individual datasets are based on at least four animals/treatment group.
**Figure S2: Nicotine‐induced effects on inhibitory neurotransmission are short‐lasting.** There were no significant effects by nicotine‐treatment on recorded sIPSCs in neither DMS (A‐D), nor DLS (E‐H), one month after cessation of nicotine exposure. Data are mean values ± SEM. n = the number of cells. Individual datasets are based on at least four animals/treatment group.
**Figure S3: Rotarod training affects neurotransmission in nAc shell.** One week of training on the rotarod significantly depressed input/output function also in nAc shell (B), but had no effect on evoked potentials in nAc core (A), CeA (C) or BLA (D). Data are mean values ± SEM. n = the number of recordings. Individual datasets are based on at least five animals/treatment group.
**Figure S4: Nicotine‐induced effects on inhibitory neurotransmission are inhibited in animals trained on the rotarod.** A, D) Potentiation of picrotoxin‐induced disinhibition was not present in nicotine‐treated animals trained on the rotarod. While a significant decrease in sIPSC frequency was found in the DMS (B, C), nicotine‐induced effects previously present in the DLS was not present after rotarod training (E, F). Data are mean values ± SEM. n = the number of recordings. Individual datasets are based on at least five animals/treatment group.
