# Supplementary material for: Nicotine‐induced neuroplasticity in striatum is subregion‐specific and reversed by motor training on the rotarod
Source: Addict Biol. 2019 Apr 10;25(3):e12757. doi: 10.1111/adb.12757 (PMC7187335; doi:10.1111/adb.12757)
Supplement: Supplementary file 1 — Figure S1. Synaptic depression induced by dopamine D2 receptor activation is not significantly modulated by nicotine or rotarod‐training. A, C) There was no effect by previous nicotine‐treatment on the responsiveness to the dopamine D2 receptor agonist quinpirole. B, D) Five days of rotarod‐training was not sufficient to modulate quinpirole‐induced synaptic depression in dorsal striatum. Data are mean values ± SEM. n = the number of recordings. Individual datasets are based on at least four animals/treatment group. Figure S2: Nicotine‐induced effects on inhibitory neurotransmission are short‐lasting. There were no significant effects by nicotine‐treatment on recorded sIPSCs in neither DMS (A‐D), nor DLS (E‐H), one month after cessation of nicotine exposure. Data are mean values ± SEM. n = the number of cells. Individual datasets are based on at least four animals/treatment group. Figure S3: Rotarod training affects neurotransmission in nAc shell. One week of training on the rotarod significantly depressed input/output function also in nAc shell (B), but had no effect on evoked potentials in nAc core (A), CeA (C) or BLA (D). Data are mean values ± SEM. n = the number of recordings. Individual datasets are based on at least five animals/treatment group. Figure S4: Nicotine‐induced effects on inhibitory neurotransmission are inhibited in animals trained on the rotarod. A, D) Potentiation of picrotoxin‐induced disinhibition was not present in nicotine‐treated animals trained on the rotarod. While a significant decrease in sIPSC frequency was found in the DMS (B, C), nicotine‐induced effects previously present in the DLS was not present after rotarod training (E, F). Data are mean values ± SEM. n = the number of recordings. Individual datasets are based on at least five animals/treatment group. [file ADB-25-e12757-s001.docx]

**SUPPLEMENTAL INFORMATION**


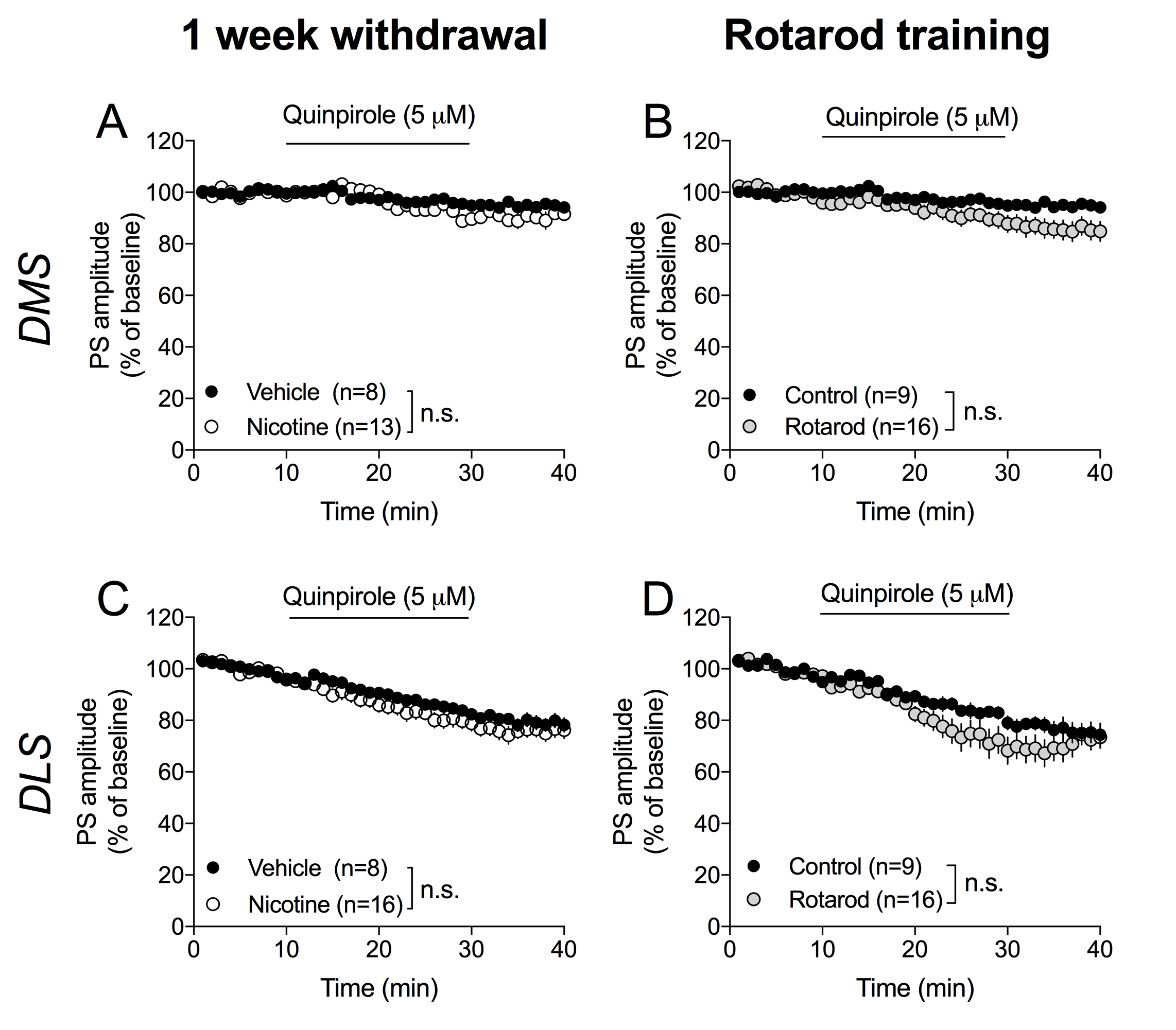


**Figure S1. Synaptic depression induced by dopamine D2 receptor activation is not significantly modulated by nicotine or rotarod-training.** A, C) There was no effect by previous nicotine-treatment on the responsiveness to the dopamine D2 receptor agonist quinpirole. B, D) Five days of rotarod-training was not sufficient to modulate quinpirole-induced synaptic depression in dorsal striatum. Data are mean values ± SEM. n = the number of recordings. Individual datasets are based on at least four animals/treatment group.


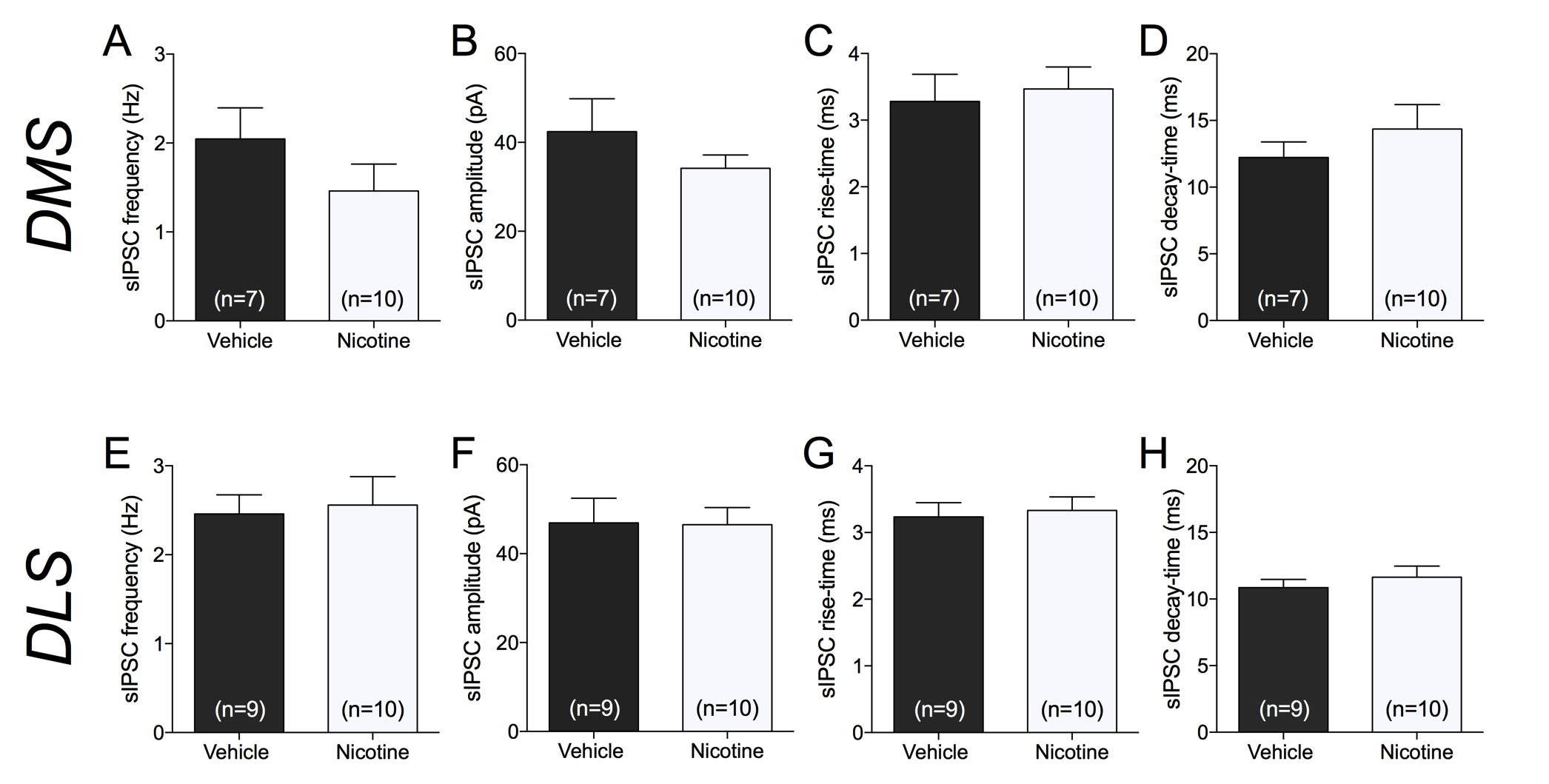


**Figure S2: Nicotine-induced effects on inhibitory neurotransmission are short-lasting.** There were no significant effects by nicotine-treatment on recorded sIPSCs in neither DMS (A-D), nor DLS (E-H), one month after cessation of nicotine exposure. Data are mean values ± SEM. n = the number of cells. Individual datasets are based on at least four animals/treatment group.

**
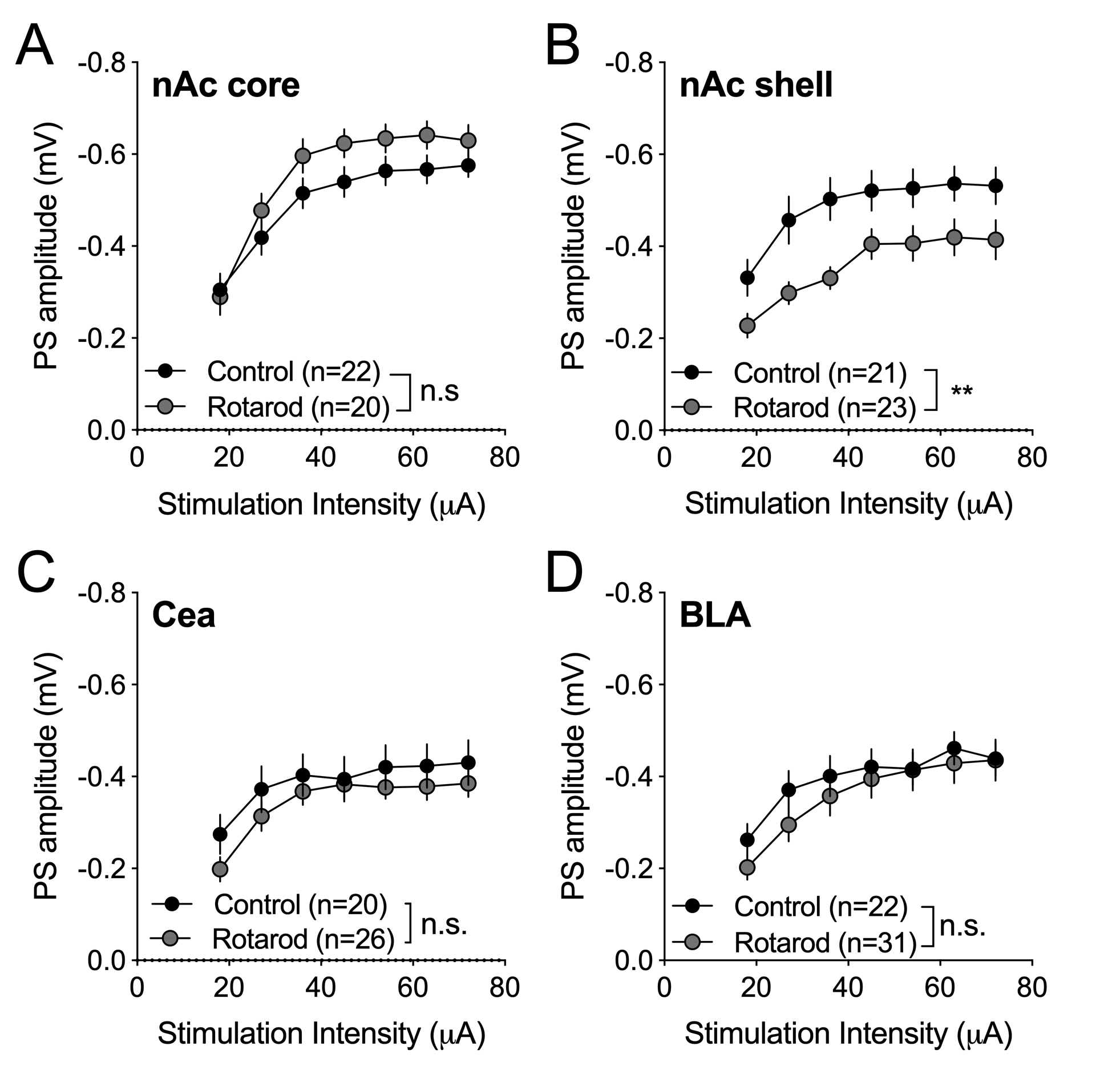
**

**Figure S3: Rotarod training affects neurotransmission in nAc shell.** One week of training on the rotarod significantly depressed input/output function also in nAc shell (B), but had no effect on evoked potentials in nAc core (A), CeA (C) or BLA (D). Data are mean values ± SEM. n = the number of recordings. Individual datasets are based on at least five animals/treatment group.


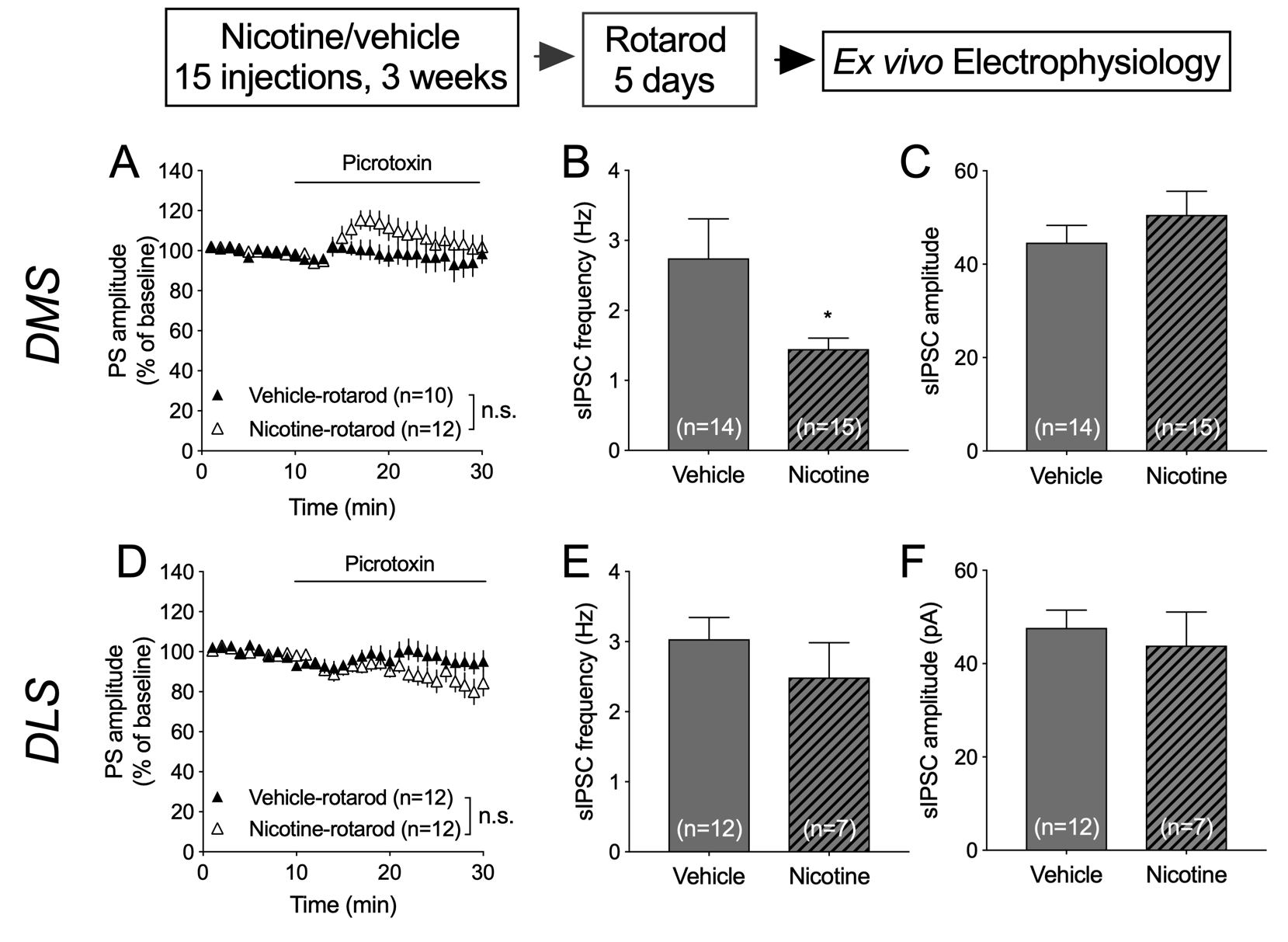


**Figure S4: Nicotine-induced effects on inhibitory neurotransmission are inhibited in animals trained on the rotarod.** A, D) Potentiation of picrotoxin-induced disinhibition was not present in nicotine-treated animals trained on the rotarod. While a significant decrease in sIPSC frequency was found in the DMS (B, C), nicotine-induced effects previously present in the DLS was not present after rotarod training (E, F). Data are mean values ± SEM. n = the number of recordings. Individual datasets are based on at least five animals/treatment group.
